# Development and validation of a novel panel of 16 STR markers for simultaneous diagnosis of β-thalassemia, aneuploidy screening, maternal cell contamination detection and fetal sample authenticity in PND and PGD/PGS cases

**DOI:** 10.1038/s41598-019-43892-2

**Published:** 2019-05-15

**Authors:** Zohreh Sharifi, Faezeh Rahiminejad, Atefeh Joudaki, Ameneh Sarhadi Bandehi, Hossein Farahzadi, Yeganeh Keshvar, Fatemeh Golnabi, Sanaz Naderi, Rasaneh Yazdani, Mehdi Shafaat, Shirin Ghadami, Maryam Abiri, Sirous Zeinali

**Affiliations:** 1 Dr. Zeinali’s Medical Genetics Laboratory, Kawsar Human Genetics Research Center, Tehran, Iran; 2 Faculty of Advanced Sciences and Technology, Department of Genetics, Tehran Medical Sciences, Islamic Azad University, Tehran, Iran; 3 Proteomics Research Center, Shahid Beheshti University of Medical Sciences, Tehran, Iran; 4 Noor Human Genetic Research Center, Tehran, Iran; 5 Department of Medical Genetics and Molecular Biology, School of Medicine, Iran University of Medical Sciences, Tehran, Iran; 6 Department of Molecular Medicine, Biotechnology Research Center, Pasteur Institute of Iran, Tehran, Iran

**Keywords:** Genetics, Haplotypes

## Abstract

Prenatal diagnosis (PND) may be complicated with sample mix-up; maternal cell contamination, non-paternity and allele drop out at different stages of diagnosis. Aneuploidy screening if combined with PND for a given single gene disorder, can help to detect any common aneuploidy as well as aiding sample authenticity and other probable complications which may arise during such procedures. This study was carried out to evaluate the effectiveness of a novel panel of STR markers combined as a multiplex PCR kit (HapScreen™ kit) for the detection of β-thalassemia, aneuploidy screening, ruling in/out maternal cell contamination (MCC), and sample authenticity. The kit uses 7 STR markers linked to β-globin gene (HBB) as well as using 9 markers for quantitative analysis of chromosomes 21, 18, 13, X and Y. Selection of the markers was to do linkage analysis with β-globin gene, segregation analysis and to perform a preliminary aneuploidy screening of fetal samples respectively. These markers (linked to the β-globin gene) were tested on more than 2185 samples and showed high heterozygosity values (68.4–91.4%). From 2185 fetal cases we found 3 cases of non-paternity, 5 cases of MCC, one case of sample mix-up and one case of trisomy 21 which otherwise may have end up to misdiagnosis. This kit was also successfully used on 231 blastomeres for 29 cases of pre-implantation genetic diagnosis (PGD) and screening (PGS). The markers used for simultaneous analysis of haplotype segregation and aneuploidy screening proved to be very valuable to confirm results obtained from direct mutation detection methods (i.e. ARMS, MLPA and sequencing) and aneuploidy screening.

## Introduction

β-thalassemia (β-thal) is the most common autosomal recessive disease in the world, particularly in the Middle East^1^. It is estimated that there are nearly 80 million carriers of β-thal worldwide. There would be approximately 60,000 affected babies born annually worldwide if no prevention program was implemented^2,3^. Carrier prevalence in Iran, with a population of about 80 million, is estimated to be 3–4 million (4–5%), with a broad spectrum of mutations^4^. Considering 1.5 million births per a year, one would expect to see birth of about 700–800 affected children, however, this number has been reduced to less than 150 since a nationwide prevention program was implemented^5^.

PND (prenatal diagnosis) is the method of choice for the prevention of new cases in carrier couples. PND has been in practice for the past several decades^4,6^ and as early as 1993 in Iran^7^. PND is a complicated process which poses misdiagnosis for various reasons including sample mix-up, non-paternity, maternal cell contamination, etc. Evaluation of the mentioned issues is essential in most PND cases and requires time and money otherwise misdiagnosis may occur.

PND usually requires invasive fetal sampling; therefore, to prevent invasive fetal sampling, preimplantation genetic diagnosis (PGD) is used as an established procedure to detect genetic disorders at the embryonic stage. PGD enables us to simultaneously check blastomeres for the disease gene alone or combined with HLA typing (using linked STR markers for haplotyping), aneuploidy detection and gender selection^8,9^.

In more than a decade, clinical validity of QF-PCR has been established as a rapid technique for aneuploidy diagnosis or exclusion of aneuploidy of chromosomes 13, 18, 21, X or Y^10^ for at-risk pregnancies as well as in PGS (preimplantation genetic screening). In most western and many other countries including Iran, every pregnant woman has to be screened for chromosomal aneuploidy^11^.

In this research, we validated a panel of multiplex-PCR based STR genotyping kit (i.e. Hapscreen kit from Genetek Biopharma, Berlin, Germany, GT) which uses STR markers closely linked to the β-globin gene to aid the diagnose of β-thal as a linkage approach for increasing the accuracy of results obtained by direct method (e.g. Sanger sequencing, MLPA or ARMS methods). According to the manufacturer’s claim, to enhance the functionality of this panel, different markers from chromosomes of 13, 18, 21, X, and Y have been added to the above panel. Addition of the mentioned markers facilitate aneuploidy screening, detecting maternal cell contamination, confirming fetal sample authenticity in addition to the diagnosis of β-thal simultaneously.

## Materials and Methods

### Samples preparation

The study was approved by the ethics committee of Kawsar Human Genetic Research Center. We confirm that all methods were performed in accordance with the relevant guidelines, regulations or protocols. All couples who had applied for PND or PGD were initially visited by a clinical geneticist and their genetic and reproductive history was determined and probable diagnostic choices were discussed.

After obtaining informed consent, different samples from chorionic villus (CV) or blastomeres were obtained by the specialists. DNA was extracted from CV samples using DNA Isolation kit (Kawsar Biotech Co., Tehran, Iran, KBC) following manufacturer’s protocol. Genomic DNA was extracted from blood samples by standard salting out method^12^.

### STR identification and selection

At initial phase, 15 STR markers linked to the β-globin gene were selected using UniSTS database and UCSC genome browser. Six of the selected STRs markers were novel using TRF(Tandem Repeats Finder)^13^. All selected STRs were tri or tetra nucleotide repeats to increase the accuracy of diagnosis and reduce the stutter artifacts. All primers were designed using Gene Runner software v3.05^14^ and synthesized and labeled commercially. The sequences of these primers are in the province of Genetek Biopharma company. Afterward, 100 unrelated healthy individuals were genotyped to assess genotype frequency for each STR marker. Finally, seven markers were chosen to use in kit manufacturing.

### Multiplex-PCR

We obtained GT-HapSceen, Aneusure (a QF PCR kit) and GT-Detector (a human DNA profiling kit) Kits form GeneTek (GeneTek Biopharma, Berlin, Germany, GT). Multiplex-PCR optimization was performed for blood samples and single cell blastomeres separately according to manufacturer’s recommendation and protocols (though the manufacturer had not suggested its use in PGD/PGS). PCR products were analyzed on 2% (w/v) agarose gel (KBC) prior to running on Genetic Analyzer 3130 XL (Thermo Fisher Scientific, US, TF) to check the quality of PCR products.

### Haplotype phasing

In order to phase haplotypes, we either used information for each marker from couples’ affected child and if there was no child then a carrier (based on CBC and A2 blood test results or confirmed mutation testing) parent of each partner was used. We have the habit in putting the mutated alleles and therefore haplotype on the left side of the bar (based on the affected child’s haplotype and pattern of inheritance, from the father or mother) and draw each allele for each individual and writing the mutation carrying allele on the left side of the bar. This will be carried out until all alleles are place in either side of the bars. However, for chromosome 13, 18, 21, X and Y we only draw alleles based on haplotypes but try to put paternal ones on the left side of the bar.

### Fragment analysis

1 µ of each PCR product was mixed with HiDi Formamide and GT-500 size standard (GT) denatured at 95 °C for 3 min and snap cooled on ice for 5 min. The denatured PCR products were then separated and detected using an ABI 3130 XL or 3500 XL Genetic Analyzer (TF). Data were analyzed using Genemapper software (TF).

### Statistical analysis

Allele frequencies for each STR loci, the power of discrimination (PD)^15^ and the power of exclusion (PE)^16^, were computed using the PowerStatsV12 Software^17^ and Gene Alex^18^. The polymorphic information content, the expected and observed heterozygosity, and the deviation from the Hardy–Weinberg equilibrium (HWE) were calculated based on the Fisher’s exact test method^15^.

## Results

### Prenatal diagnosis and PGD outcomes

Couples who are either confirmed carriers of β-thal or are suspected of being carrier of β-thal are counseled and are referred for PND. Mutation detection is usually carried out by testing both couple or their affected child by either ARMS method^19,20^, Sanger sequencing or MLPA. We also would haplotype all cases of confirmed β-thal using RFLP or SNP markers linked to β-globin gene^21–23^.

For haplotyping, we would use phasing information from the couple, their affected child (if present or available) and/or their parents. Haplotyping have been an essential part of our PND for thalassemia and most other diseases. When the Hapscreen kit became available, we switched to Hapscreen for almost every cases of PND. Therefore, every PND is performed using both direct mutation detection and haplotyping.

**Figure 1:**
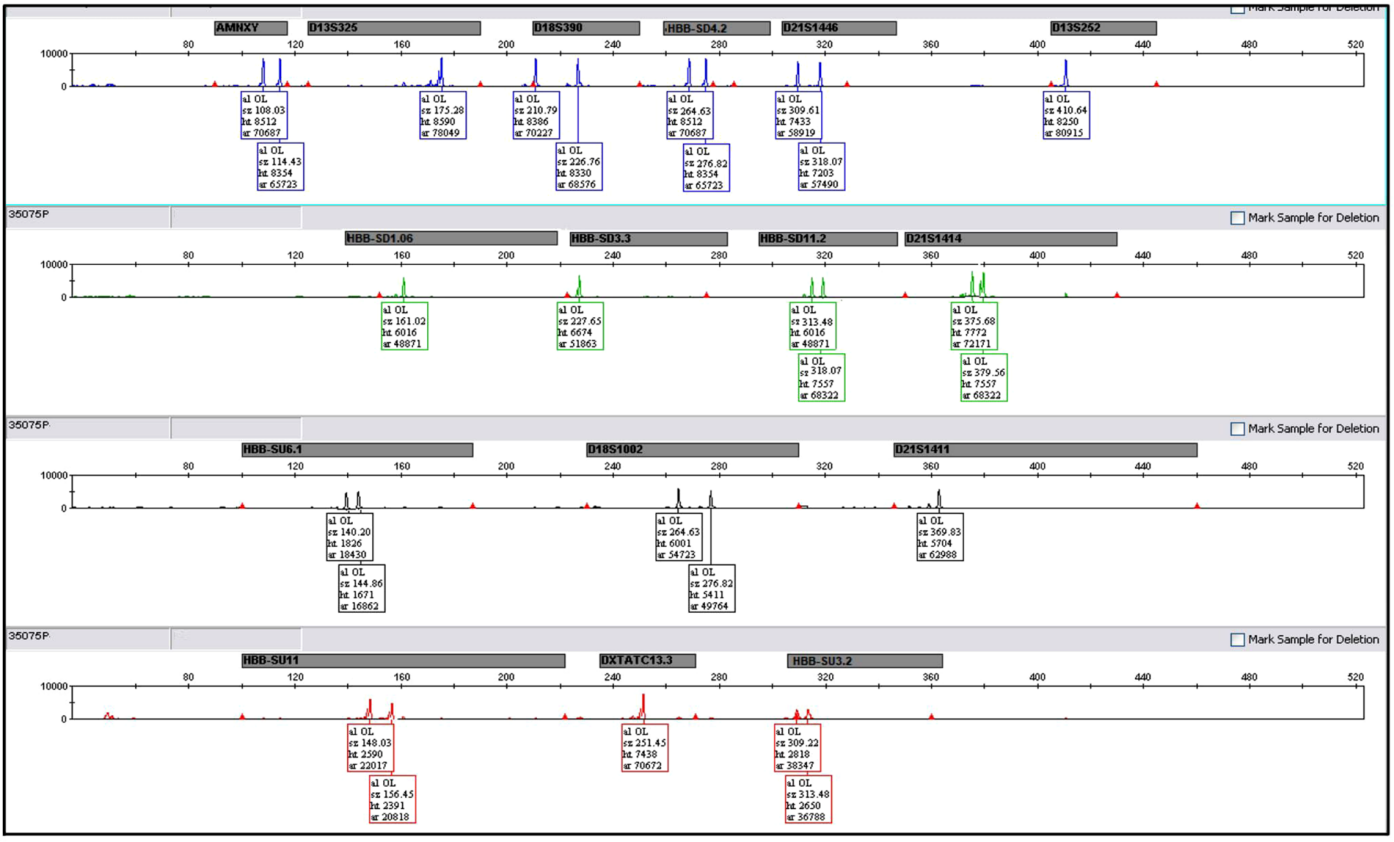
Capillary electrophoresis result of a multiplex PCR for 16 selected STR markers. The name of each marker is written above the peaks. The data is generated using HapScreen HBB. These types of data have been used to draw pedigree with haplotypes (e.g. Figs 2, 3 and 5).

**Figure 2:**
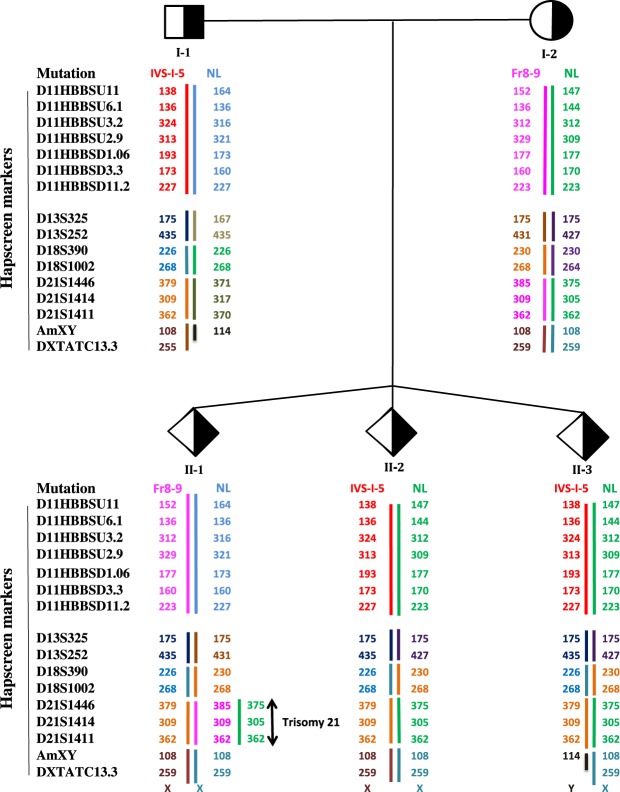
A β–thalassemia case with triplet pregnancy. I-1 (the father) carries IV S-I-5 and I-2 (the mother) carries Fr8–9 mutations. II-1 is a female carrier fetus affected with trisomy 21. II-2 and II-3 are carrier female and male fetuses respectively. The last two fetuses are very similar in their haplotypes. The only difference is in Amelogenin (AmXY) marker which helped us in differentiating these two fetuses from each other. Therefore, confirming sample authenticity and MCC is ruled out for all three fetuses. “NL” means normal or wild type allele.

**Figure 3:**
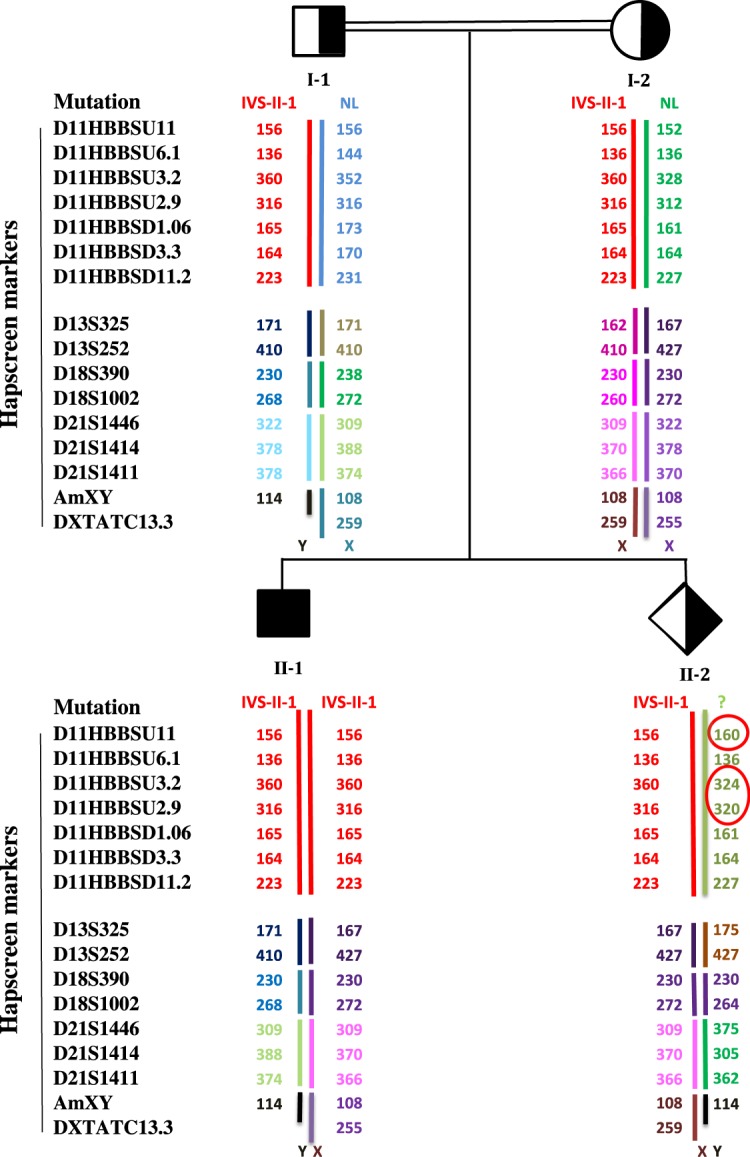
A β –thalassemia PND case from CVS which demonstrates sample authenticity dispute. It shows that the fetus (II-2) does not share some of his haplotypes from his father (lacking shared haplotype [with mutation] from his father if the red haplotype is from the mother). Discrepancy in other marks on different chromosomes (13, 18 and 21) showed this fetus is not the biological child of the father. “NL” means normal or wild type allele.

More than 2185 fetal cases of β-thal have been diagnosed using the above procedures. From these, 528 fetal samples were homozygous normal, 1105 heterozygous carriers and 552 were affected and all of them were referred for therapeutic abortions. On the other hand, 949 out of 2185 cases were screened for aneuploidies using Hapscreen kit. The PND outcomes were 227 homozygous normal, 494 heterozygote and 228 affected fetuses. From 949 fetal cases, 917 were singleton, 31 were twin and one case was triplet. Among 2185 fetal cases we identified 5 cases of non-paternity, 3 cases of sample mix-up (misdiagnosis was prevented based on Hapscreen results), 3 cases of maternal cell contamination (MCC) and one case of trisomy 21 (suggested by Hapscreen result and confirmed by QFPCR). Some of these cases are shown in Figs 1–3 and elaborated below when figures are being explained.

In total, we analyzed more than 238 blastomeres from 29 PGD cases. Our analysis showed that 51 blastomeres were homozygous normal, 82 were carriers, 55 were affected (not transferable) and the rest (50 blastomeres) did not produce any result. This may have been either the sample had been deliberately blanked by the IVF center, or no cells had been transferred into the tubes (either the cell had been lysed before transferring or had been released onto the tubes wall).

In total 124 embryos were transferable, though in few cases the parents had decided only to transfer homozygous normal cells. To date 11 pregnancies had occurred, two was miscarried, 3 are ongoing and 5 healthy babies have been born.

### Heterozygosity assessment of the markers

Allele frequencies and heterozygosity of 15 β-globin STR markers were obtained by genotyping 100 unrelated healthy individuals prior to use them in haplotyping fetal samples. In total, 133 different alleles were detected (data is shown in Table 1).

**Table 1:** Statistical findings of HBB STR markers.

loci	D11HBBSD0.4	D11HBBSD3.3	D11HBBSU2.9	D11HBBSU6.1	D11HBBSD11.2	D11HBBSU11.0	D11HBBSU8.7	D11HBBSU0.6	D11HBBSD4.2	D11HBBSU15.6	D11HBBSD3.2	D11HBBSD12.7	D11HBBSD15.6	D11HBBSD1.06	D11HBBSU3.2
Size range	290–325	140–200	300–360	120–180	210–290	125–210	305–348	238–275	264–292	206–295	337–375	196–224	166–194	140–220	300–384
Matching Probability	0.109	0.067	0.171	0.15	0.15	0.081	0.13	0	0.069	0.083	0.068	0.108	0.16	0.068	0.094
Expressed as 1 in…	9.2	14.9	5.9	6.7	6.7	12.3	11.5	6.6	14.5	12	15.1	9.1	6.1	14.8	10.6
PD*	0.857	0.924	0.868	0.866	0.761	0.908	0.921	0.771	0.925	0.917	0.881	0.854	0.865	0.932	0.906
PIC	0.74	0.78	0.64	0.69	0.75	0.75	0.77	0.76	0.82	0.9	0.77	0.75	0.62	0.89	0.91
PE**	0.215	0.413	0.528	0.599	0.407	0.543	0.523	0.551	0.648	0.463	0.421	0.217	0.525	0.825	0.928
Typical Paternity Index	1.06	1.61	2.09	2.5	1.59	2.17	2.54	1.45	2.88	1.8	1.72	1.05	2.05	5.83	14
No. of Alleles	8	9	6	6	5	11	12	5	8	15	9	8	6	11	14
Het obs	0.422	0.724	0.724	0.737	0.557	0.684	0.692	0.513	0.770	0.788	0.701	0.518	0.785	0.909	0.964
Het exp	0.744	0.787	0.733	0.726	0.590	0.764	0.782	0.575	0.821	0.901	0.68	0.615	0.734	0.932	0.917
P HWE	P < 0.001	P < 0.001	P < 0.001	P < 0.001	P < 0.001	P < 0.001	P < 0.001	P < 0.001	P < 0.01	P < 0.001	P < 0.001	P < 0.001	P < 0.001	P < 0.001	P < 0.001

PD stands for power of discrimination, PE: power of exclusion, PIC: polymorphic information content, Het obs: observed heterozygosity, Het exp: expected heterozygosity, PHWE: significane of deviation from Hardy–Weinberg equilibrium.

The observed range of allele frequencies varied. According to our study, D11HBBSU3.2 marker had the highest heterozygosity (96.4%) among the 15 studied STR markers. D11HBBSD1.06 marker with 90.9% frequency rate was the second most heterozygous marker. The allele size ranges and heterozygosity of STR loci are listed in Table 1. The PD ranges were from 0.76 to 0.93 for D11HBBSD11.2 and D11HBBSU3.2 loci respectively. Estimating allele and genotype frequencies showed no deviation from the Hardy–Weinberg equilibrium (P < 0.001). Other statistical findings are shown in Table 1. Allele frequencies for QF-PCR part of the kit were not calculated, though are available since they are generated simultaneously in every test (Fig. 1) and are available elsewhere since they are commonly used in most commercially available QFPCR kits including AneuSure (GT)^24–28^.

### Markers used in PND and PGD

Since the initial use of the β-globin HapScreen kit, we have been using it routinely in almost all cases of PNDs for β-thal. It has been extremely valuable to track the disease gene segregation and also confirm results obtained by other methods (e.g., ARMS or Sanger sequencing), paternity, sample authenticity, or even uniparental disomy^29^.

Also it has been shown to be extremely valuable when the disease is confirmed by other methods (e.g. CBC, HBA2 level and clinical presentation). Indeed in several cases the initial investigation of the β-globin gene by sequencing or MLPA had not resulted in identifying a definite causal mutation in several families with CBC and HbA2 levels similar to those of β-thal carriers, who had been referred to our lab for either PND or PGD, despite of our center being the most experienced lab in the country^5^. Therefore, for the purpose of PND, indirect approaches such as using RFLP, SNP or STR markers can be used^21,30^. In addition, 29 other families were considered as a candidate for PGD, with a medical history of at least two abortions due to fetuses being diagnoses as affected with β-thal and parents’ desire was not to go through natural pregnancy which necessitates PND.

Our policy for carrying our PND or PGD is that the haplotypes from family members should be determined to track the mutant allele even before fetal sampling and in every case of PGD before starting IVF procedure.

Below several cases have been given to clearly demonstrate our approaches. For example, Fig. 2 shows results of haplotyping for a β–thal family with a triplet pregnancy. We knew that the personI-1 (the father) carries IVS-1-5 mutation and I-2 (the mother) carries a frameshift 8–9 (Fr8–9) mutation. Haplotyping in the pedigree shows that II-1 is a female carrier fetus affected with trisomy 21. II-2 and II-3 are female and male carrier fetuses respectively. The last two fetuses are very similar in their haplotypes. The only difference is in their Amelogenin (AmXY) region which helped us to differentiate these two fetuses from each other (confirming sample not being mixed-up). Other confirmatory test also confirmed sex typing results. Sample authenticity is confirmed by shared alleles between the parents and fetuses and also MCC is ruled out for all three fetuses.

**Figure 4:**
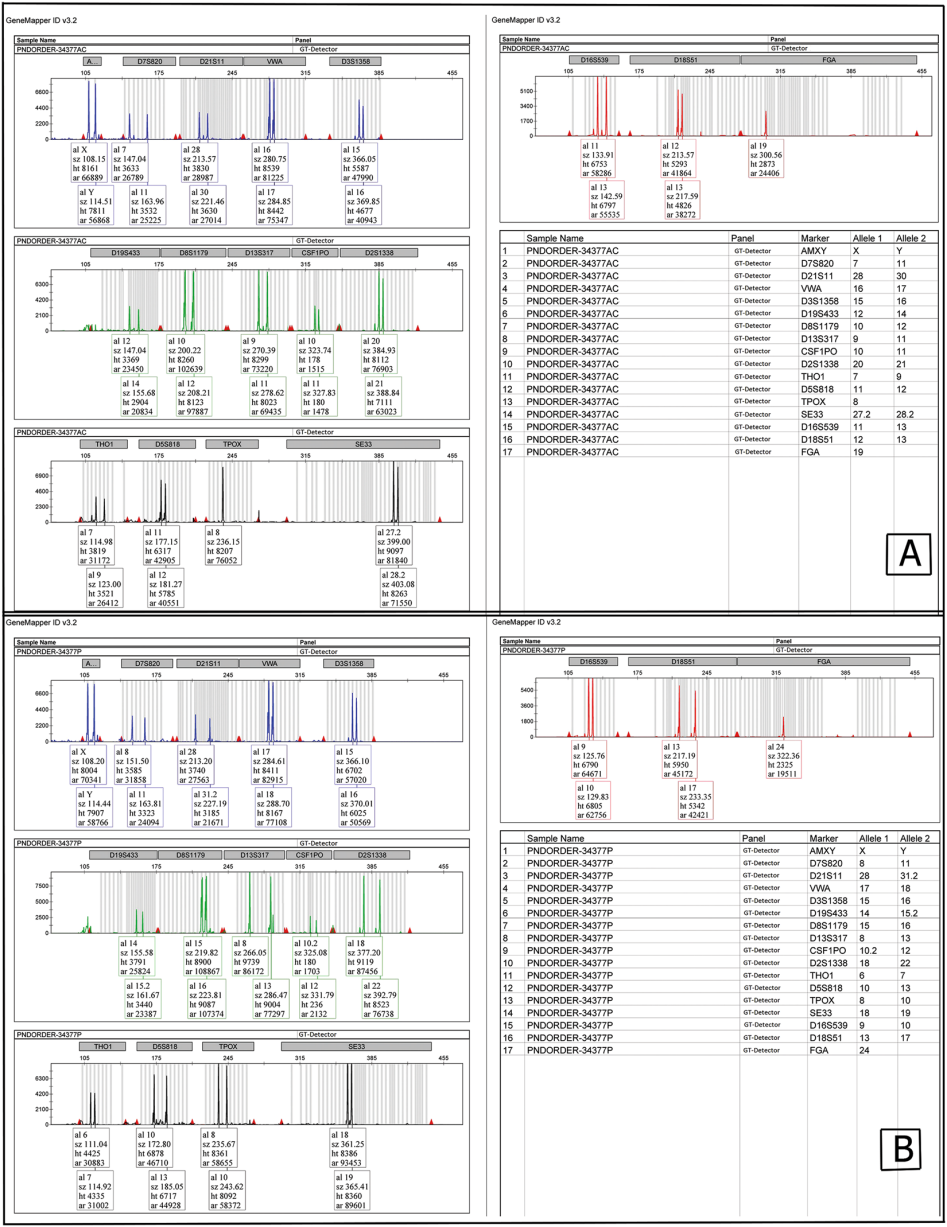
Paternity test result, (**A**) fetus DNA profile using GT-detector, (**B**) father’s DNA profile. These two have several unshared markers.

In Fig. 3 we have another case of β-thal. In this case, haplotyping confirmed non-paternity. Since the carrier couple shares the same mutation and haplotype, the fetus shares one of the haplotypes but the other one is not present in either the mother or the father, confirming non-paternity but sample authenticity since it had maternal haplotype. No chromosomal aneuploidy was observed. We do not have the habit in informing the parents in non-paternity cases, though no official rule is in place to follow. Having extra markers from the Aneusure QF-PCR kit (GT) included in this panel provided us the opportunity to rule out or confirm sample authenticity and non-paternity. For further confirmation, paternity testing was performed using GT-Detector kit (GT). As presented in Fig. 4, the paternity was rejected by this approach as well.

**Figure 5:**
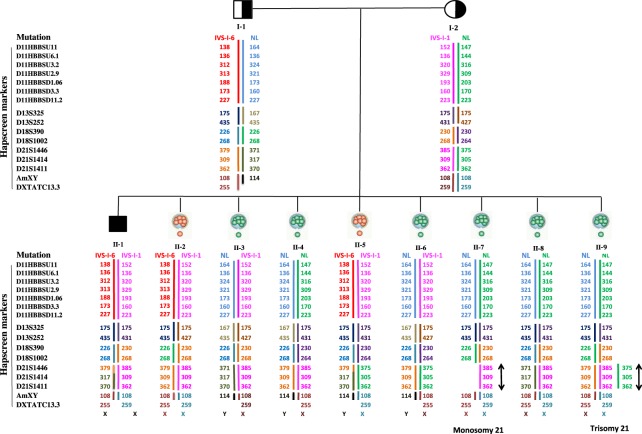
Schematic representation of a PGD result for beta-thalassemia and aneuploidy. As it is shown the assigned haplotypes are shown underneath each blastomere, parents and the affected child. The order of corresponding STR markers are shown on left. I-1 (father) is a carrier of IVSI-6 mutation and I-2 (mother) is carrier for IVSI-1. II-1 is the affected child of the family. II-2 is the female blastomere affected with β-thal, II-3 is a male who carriers IVSI-1 mutation, II-4 is normal male, II-5 is affected male, II-6 is carrier male, II-7 is normal female but who is affected with monosomy 21, II-8 is a normal female, II-9 a is normal female but is affected with trisomy 21. The conclusion is that II-3, II-4, II-6 and II-8 are not affected and were reported to the IVF center to decide which to be transferred.

The final example is a case of PGD for β-thal which is shown in Fig. 5. II-7 and II-9 blastomeres are not affected with thalassemia but they suffer from monosomy of 21 and trisomy of 21 respectively. Therefore, the only four transmissible blastomeres were II-3, II-4, II-6 and II-8.

## Discussion

β-thal is the most common hematological disorder in the Middle Eastern countries^31^. National Program for the Prevention of Thalassemia has been in effect since 1997 in Iran^5,6^. Since then, many different types of mutation have been diagnosed^32^. However, there still exist cases with hematological parameters indicating β-thal but no mutation can be found despite using all available techniques like sequencing, gap PCR, MLPA and NGS^33,34^.

We showed that using STR markers can be very helpful in several ways for performing either PGDs^35^ or in special cases of PNDs where hematological indices indicate that the parents are carrier/s of β-thal^36^, even when no mutation can be found using techniques like whole gene sequencing or MLPA^37^. In addition, haplotype analysis can be helpful when there is a time constraint to find the mutation and even confirming direct mutation analysis findings^38^.

Although linkage analysis is very practical, it has some limitations such as some markers may not be informative in some families or no other number of the family is available for genotyping^39,40^. The performance of homozygosity mapping is highly depending on markers heterozygosity. Another limitation is that it can’t be used if involvement of beta-globin gene is not 100% confirmed.

Regarding aborting affected fetuses, there is a religious decree and also a law allowing legal abortion in Iran. The law has been in effect since 2006 and religious decree since 1996^4^. For PGDs we have the habit to ask the family to undergo PND at 11–16 weeks’ gestation to avoid any misdiagnosis either at molecular stage or at embryo transfer. To date we have not had any misdiagnosis for beta-thalassemia and also if a family refuses to do PND, we do not force them. Regarding embryo transfer, in most PGD cases (except in few hemophilia A or B cases in which families have insisted in not transferring carrier female embryos) we suggest that both homozygous normal and heterozygote carriers’ embryos be transferred.

We showed that this newly released HapScreen kit can overcome these problems since different tetra-nucleotide repeat markers with a high rate of heterozygosity have been added into the kit to reduce the rate of stutter artifacts which is usually seen in dinucleotide repeat units^41^. In addition, to minimize the possibility of meiosis recombination, physical distances of newly selected markers to the gene have been chosen to be less than 1Mbp. To further assess the suitability of the markers, allele frequency and heterozygosity of each STR marker were calculated in at least 100 unrelated individuals.

Using multiplex-PCR in linkage analysis, not only requires less time, reagents and labor work; it also decreases the sampling error rate. More importantly, it compensates the problem of insufficiency of blastomeric DNA used in PGD. Determining sample authenticity is an important issue in PND and PGD. There is a 50% similarity of haplotypes between the fetus and the mother; therefore, it is of great significance to confirm that the sample belongs to the fetus and it is not merely a result of maternal cell contamination. By adding nine markers from different chromosomes in addition to seven markers linked to the *HBB* gene, it seems very unlikely that two individuals would have the same haplotype.

Errors can arise in every stage of CVS or amniocentesis in twin pregnancies. Therefore, the kit has a tool to differentiate between fetuses in twin or multiple pregnancies or between samples which have under gone PND testing (i.e. parental, sibs and fetuses’), to reduce the rate of errors. By choosing multiple markers from different chromosomes (i.e. chromosomes 6, 21, 18, 13, X and Y), the chance of observing the same haplotype in different individuals would be reduced. Therefore, this important feature would be very helpful to rule out maternal contamination, sampling errors, investigating sample authenticity and differentiation of fetal samples in multiple pregnancies. It can also confirm triploidy or uniparental disomy.

To achieve these goals, the manufacturer could have selected these markers from markers that are used in different human identification kits (e.g. GT-Detector human identification kit, GT), but they have preferred to choose them from chromosomes that are major cause of common aneuploidies^42^. In addition, to achieve the above mentioned goals, these markers allow early screening for common aneuploidies and sex determination in fetal samples and blastomeres cells.

We have been able to use HapScreen in several hundreds of our PND and PGD cases with no shortcomings and we think it adds credibility to every PND and PGD and also increases diagnostic accuracy and peace of mind in a discipline prone to misdiagnosis.

## Data Availability

The authors confirm that the data supporting the findings of this study are available within the article and more information that support the findings of this study will be available on request from the corresponding.
